# Investigation of the Chemical Composition, Antihyperglycemic and Antilipidemic Effects of *Bassia eriophora* and Its Derived Constituent, Umbelliferone on High-Fat Diet and Streptozotocin-Induced Diabetic Rats

**DOI:** 10.3390/molecules27206941

**Published:** 2022-10-16

**Authors:** Abdulaziz K. Al Mouslem, Hany Ezzat Khalil, Promise Madu Emeka, Ghallab Alotaibi

**Affiliations:** 1 Department of Pharmaceutical Sciences, College of Clinical Pharmacy, King Faisal University, Al-Ahsa 31982, Saudi Arabia; 2 Department of Pharmacognosy, Faculty of Pharmacy, Minia University, Minia 61519, Egypt; 3 Department of Pharmaceutical Sciences, College of Pharmacy, Shaqra University, Shaqra 11961, Saudi Arabia

**Keywords:** *Bassia eriophora*, diabetes mellitus, STZ, umbelliferone, high-fat diet

## Abstract

This study was designed to investigate the chemical profile, antihyperglycemic and antilipidemic effect of total methanolic extract (TME) of *Bassia eriophora* and isolated pure compound umbelliferone (UFN) in high-fat diet (HFD)- and streptozotocin (STZ)- induced diabetic rats. TME was subjected to various techniques of chromatography to yield UFN. Diabetes was induced after eight weeks of HFD by administration of STZ (40 mg/kg) intraperitoneally, and experimental subjects were divided into five groups. The diabetic control showed an increase in levels of blood glucose throughout the experiment. Treatments were initiated in the other four groups with glibenclamide (GLB) (6 mg/kg), TME (200 mg/kg and 400 mg/kg) and isolated UFN (50 mg/kg) orally. The effect on blood glucose, lipid profile and histology of the pancreatic and adipose tissues was assessed. Both 200 and 400 mg/kg of TME produced a comparably significant decrease in blood glucose levels and an increase in insulin levels with GLB. UFN began to show a better blood sugar-lowering effect after 14 days of treatment, comparatively. However, both 400 mg/kg TME and UFN significantly returned blood glucose levels in diabetic rats compared to normal rats. Analysis of the lipid profile showed that while HFD + STZ increased all lipid profile parameters, TME administration produced a significant decrease in their levels. Histopathological examinations showed that treatment with TME and UFN revealed an improved cellular architecture, with the healthy islets of Langerhans and compact glandular cells for pancreatic cells distinct from damaged cells in non-treated groups. Conversely, the adipose tissue displayed apparently normal polygonal fat cells. Therefore, these results suggest that TME has the potential to ameliorate hyperglycemia conditions and control lipid profiles in HFD + STZ-induced diabetic rats.

## 1. Introduction

Diabetes mellitus (DM), a metabolic disorder accompanied by abnormal glucose metabolism, is one of the challenging health issues worldwide [1]. According to International Diabetes Federation (IDF), the global prevalence of DM in 2021 was estimated to be found in 537 million adults with approximately 6.7 million deaths resultant. It is anticipated that the incidence number of DM will increase to 643 million in 2030 and 784 million in 2045, according to a recent report [2]. Moreover, deficiency and/or resistance to insulin triggers metabolism disturbances of carbohydrates, proteins, and lipids, which have been linked to type 2 DM [1,3,4]. Numerous reports have established an association between type 2 DM and dyslipidemia. This well documented phenomenon provoked several complications that include microvascular damages and dysfunctions of multiple organs such as the central nervous system, the kidneys, and the heart [3,5]. In addition, 90% of diagnosed DM disease are type 2 cases, which are therapeutically managed by oral hypoglycemic agents [2]. However, these available agents have fair glycemic and lipid control, but also serious side effects such as hypoglycemia and diabetic ketoacidosis [6,7]. Therefore, researchers have shown a lot of interest in discovering alternative therapies that will help in managing this common health problem with fewer side effects [8,9].

The use of natural products in the development of treatments for chronic diseases, such as DM, is ancient in human history [10]. Evaluation of herbal plants and their derivatives with biological activities against DM has been reported by various workers [11,12]. Hence, scientific elucidation of a good candidate, might anticipate a clinical success in managing DM with little or no complications. Numerous reports have been published showing that several natural products contain bioactive constituents with strong curative properties against many illnesses such as inflammation, infection and DM [13,14,15]. Saudi Arabia is known as having one of the richest, most biodiverse floras around the Arabian Gulf and contains hundreds of species of medicinal plants [16,17]. Several of these plants have been reported to possess traditional medicine values such as antimicrobial, anti-inflammatory, and analgesic activities [18,19,20,21]. Reported studies have shown that several medicinal plants found in the Gulf region of Saudi Arabia, possess antidiabetic activities [22,23,24].

*Bassia eriophora* (*B. eriophora*) (Family: *Chenopodiaceae*) is a plant that grows naturally in the Eastern Province of Saudi Arabia, and is known as gteena [25]. Documented evidence shows that *B. eriophora* has been used in folk medicine in the treatment of Alzheimer’s disease, alopecia and gingivitis in Iran [26], whereas in Saudi Arabia, it is commonly used in the treatment of renal and rheumatic diseases, and its white fibers are used as cotton stuffing [26,27,28,29].

Pharmacologically, *B. eriophora* has been reported to have analgesic, antipyretic, antioxidant, antirheumatic, antimicrobial, anti-inflammatory, wound-healing and protein kinase-inhibitory properties [25,26,27,28,30,31,32]. Chemical composition of *B. eriophora* is reported to contain flavonoids: luteolin, acacetin-7-*O*-β-d-glucoside, diosmin, kaempferol-3-*O*-rutinoside and rutin [30,31]. Based on the available literature, there is no report on *B. eriophora* antidiabetic or hypolipidemic activities. Hence, these findings encouraged us to carry out the current study. Therefore, this study aims to investigate the chemical composition, antidiabetic and antilipidemic potentials of *B. eriophora* in high-fat diet streptozotocin (STZ)-induced diabetic rat model. 

## 2. Results

### 2.1. Isolation and Identification of Major Secondary Metabolites

**Figure 1:**
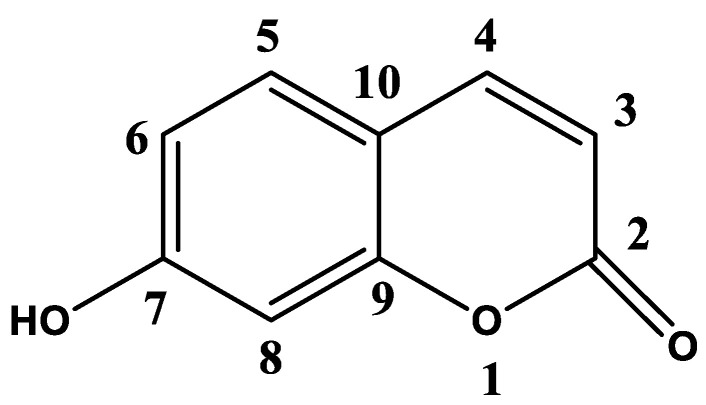
Structure of pure isolated umbelliferone (UFN).

Total methanolic extract (TME) of air-dried powdered plant material (50 g) was subjected to various and repeated chromatographic techniques, as described previously [33], to yield a pure compound, umbelliferone (UFN) [34,35]. The structure was elucidated by inspection of 1D and 2D-NMR spectroscopic data including ^1^H, ^13^C, DEPT, HMQC and HMBC (Figure 1, Supplementary Materials [S1–S6]). Results were compared with those available in the literature.

Umbelliferone: ^1^H NMR (400 MHz, DMSO-*d_6_*): δ 10.58 (br.s, OH), 7.91 (1H, d, J = 9.4 Hz, H-4), 7.51 (1H, d, *J* = 8.4 Hz, H-5), 7.78 (1H, d, *J* = 8.4 Hz, H-6), 6.71 (1H, s, H-8), 6.19 (1H, d, *J* = 9.4 Hz, H-3); ^13^C NMR (100 MHz, DMSO-*d_6_*): δ 161.26(C-7), 160.40 (C-2), 155.46 (C-9), 144.43 (C-4), 129.63 (C-5), 113.07 (C-6), 111.35(C-3), 111.23 (C-10), 102.13 (C-8).

### 2.2. Effect of TME of B. eriophora and Isolated UFN on Blood Glucose Levels

**Figure 2:**
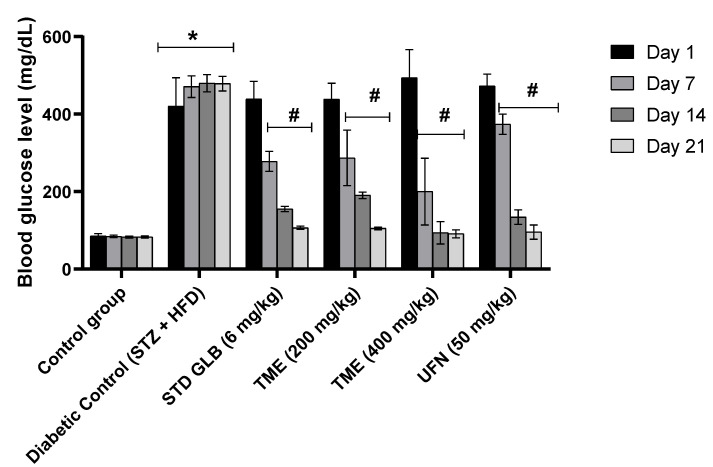
Effect of TME (200 and 400 mg/kg) of *B. eriophora* and UFN (50 mg/kg) on the blood glucose level. Data are represented as mean ± SD, *n* = 6. * represents a significant difference between the control group and the diabetic control group. Whereas # represents a significant difference between the diabetic control group with all the treatment groups of TME and UFN. STZ; streptozotocin, STD GLB; standard glibenclamide, TME; total methanol extract, UFN; umbelliferone.

Blood glucose levels in normal control and STZ-induced diabetic rats of the treatment groups for day 1, day 7, day 14, and day 21 after the STZ administration are shown in Figure 2. Following STZ treatment, GLB, a standard drug (an insulin secretagogue), (6 mg/kg), TME (200 and 400 mg/kg) and UFN, the isolated compound, (50 mg/kg) were used as treatments in the different groups. Injection of STZ at 40 mg/kg with HFD showed a significant increase (*p* < 0.001) in blood glucose levels compared to control rats. On day 7, treatment with TME 400 mg/kg produced a remarkable decrease (*p* < 0.001) in blood glucose levels amongst all the five treatment groups. It even displayed a better reduction compared to the GLB-treated group. Additionally, on day 14, it showed also a better reduction profile in blood glucose levels than all the other treatment groups, with a significant decrease (*p* < 0.001) compared to the increase in blood glucose level induced by STZ and HFD. However, the GLB-treated group showed a non-significant decrease in blood glucose level on day 14 in comparison with TME 400 mg/kg group. On day 21 of treatment, both doses of TME- and UFN-treated groups yielded results comparable to the treatment with GLB. Therefore, the use of TME of *B. eriophora,* according to obtained results, clearly shows a potential lowering effect on blood glucose levels (Figure 2).

### 2.3. Effect of TME of B. eriophora and Isolated UFN on Lipid Profile

**Figure 3:**
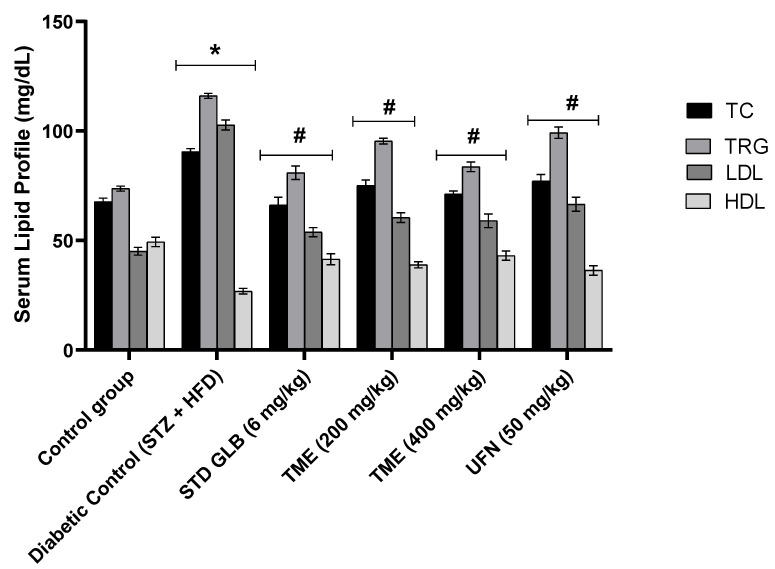
Effect of TME (200 and 400 mg/kg) of *B. eriophora* and UFN on serum lipid profile. Values are represented as mean ± SD, *n* = 6. * represents a significant difference between the control group and the diabetic control group. Whereas # represents a significant difference between the diabetic control group with all the treatment groups of TME and UFN. TC; total cholesterol, TRG; triglycerides, LDL; low-density lipoprotein, HDL; high-density lipoprotein, STZ; streptozotocin, STD GLB; standard glibenclamide, TME; total methanol extract, UFN; umbelliferon.

Serum lipid profiles in normal control and STZ-induced diabetic rats and groups treated with GLB (6 mg/kg), TME (200 and 400 mg/kg), and UFN (50 mg/kg), at the end of the experiment, are shown in Figure 3. Our results show that injection of STZ at 40 mg/kg with HFD significantly increased (*p* < 0.05) most of the serum lipid parameters compared to the control. However, the TME 400 mg/kg-treated group showed a similar lowering effect with the GLB-treated group for both total cholesterol (TC) and triglycerides (TRG). Additionally, the effects of UFN and the TME 200 mg/kg on the lipid profile of STZ- and HFD-induced hyperlipidemia were similar. All treatment groups significantly (*p* < 0.05) improved high-density lipoprotein (HDL) levels compared to the diabetic control group. Taken together, both TME doses and UFN treatments significantly decreased the increase in lipid profile induced by STZ + HFD.

### 2.4. Effect of TME of B. eriophora and Isolated UFN on Insulin Levels and Insulin Resistance

**Figure 4:**
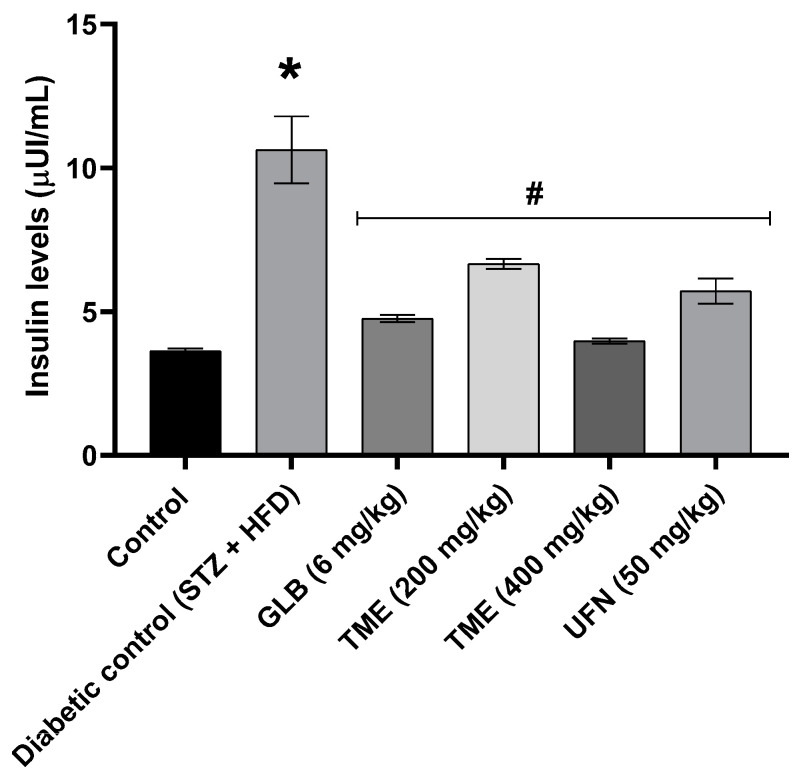
Effect of TME (200 and 400 mg/kg) of *B. eriophora* and isolated UFN on fasting insulin levels. Values are represented as mean ± SD, *n* = 3. * represents a significant difference between the control group and the diabetic control group (STZ + HFD). Whereas # represents a significant difference between the diabetic control group with all the treatment groups of TME and UFN. HDL; high-density lipoprotein, STZ; streptozotocin, STD GLB; standard glibenclamide, TME; total methanol extract, UFN; Umbelliferone.

**Figure 5:**
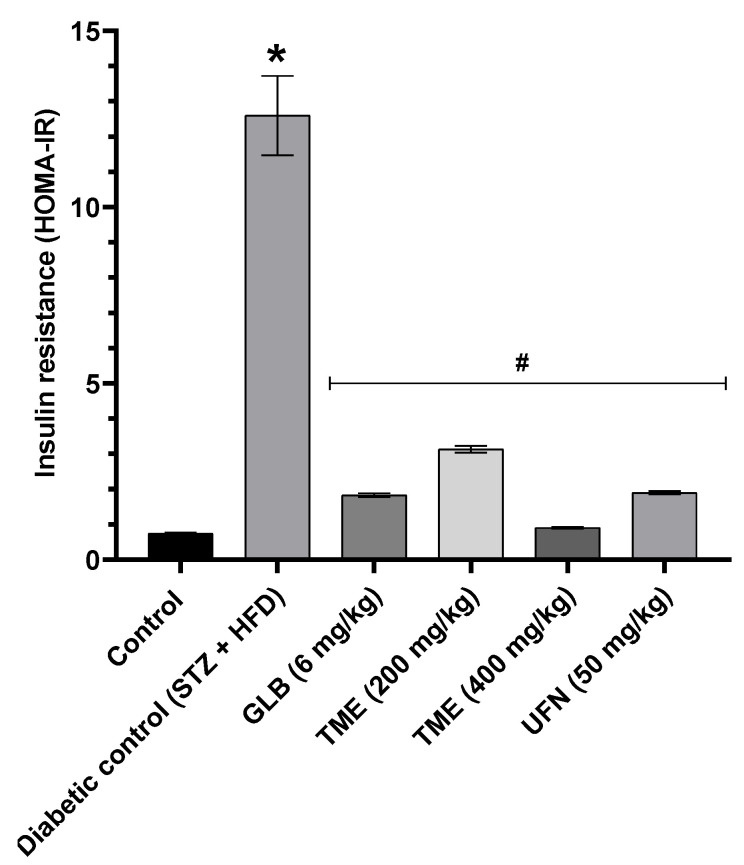
Effect of TME of *B. eriophora* and isolated UFN on insulin resistance (HOMA-IR index). Values are represented as mean ± SD, *n* = 3. * represents a significant difference between the control group and the diabetic control group (STZ + HFD). Whereas # represents a significant difference between the diabetic control group with all the treatment groups of TME and UFN. HDL; high-density lipoprotein, STZ; streptozotocin, STD GLB; standard glibenclamide, TME; total methanol extract, UFN; Umbelliferone HOMA-IR; Homeostatic Model Assessment-Insulin Resistance.

Results showed that serum insulin level was significantly (*p* < 0.001) increased after 3 weeks of treatment amongst the diabetic control (STZ + HFD) rats, as shown in Figure 4. However, treatment with TME (200 and 400 mg/kg) and UFN (50 mg/kg) reduced serum insulin levels significantly (*p* < 0.001) compared to the diabetic control group. In addition, treatment with TME (200 and 400 mg/kg) also improved insulin sensitivity significantly (*p* < 0.001) by decreasing Homeostatic Model Assessment-Insulin Resistance (HOMR-IR) in the treated rats as shown in Figure 5. Moreover, results revealed that TME acted in a dose-dependent manner. In addition, 400 mg/kg of TME effect on fasting insulin level showed no significant difference when compared with STD GLB, an insulin secretagogue. The lower dose of TME appeared to have a similar effect on insulin secretion compared to UFN, as shown in Figure 4.

### 2.5. Histopathological Analysis of Pancreatic Tissue of Diabetic Rats with TME (200 and 400 mg/kg) of B. eriophora and UFN Treatments

**Figure 6:**
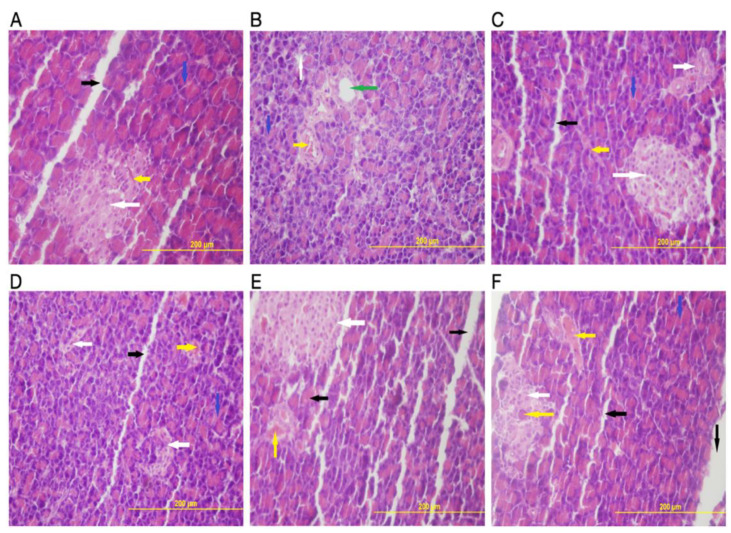
Photomicrographs of pancreatic tissue of controls and treated experimental animals. (**A**) The control group showed a healthy islet of Langerhans (endocrine cells) (white arrow), a compact glandular cells-exocrine portion with basal nuclei (blue arrow), and blood vessels (yellow arrow). (**B**) Diabetic group (STZ + HFD) showing damaged endocrine cells (white arrow), enlarged interlobular duct (green arrow), disturbed glandular cells with dilated interlobular blood vessels (yellow arrow) and perilobular fibrosis (blue arrow). (**C**) STD GLB group showing recovered endocrine cells (white arrow), with blue arrows indicating near-normal glandular cells and black arrows showing intralobular duct. (**D**) Treatment with 200 mg/kg TME showed recovering endocrine cells still having perilobular fibrosis, but not as the diabetic control group. (**E**) represents 400 mg/kg TME with recovered endocrine cells (white arrow), and blood vessels (yellow arrow) with a near-normal intralobular duct. (**F**) shows treatment effects with UFN closely similar to that produced by 400 mg/kg of TME treatment. H&E ×400. STZ; streptozotocin HFD; high-fat diet, STD GLB; standard glibenclamide, TME; total methanol extract, UFN; umbelliferone.

H&E-stained microscopic images of pancreatic sections from experimental rats after the induction of diabetes by STZ and HFD, compared to normal control and all treatment groups, are shown in Figure 6. Photomicrograph of the normal control group with healthy islets of Langerhans (endocrine cell) and with compact and intact glandular cells (representing the exocrine cells) is shown in (Figure 6A). Conversely, (Figure 6B) shows the diabetic control group with a damaged islet of Langerhans with diffused glandular cells and perilobular fibrosis compared to the normal control group. The exocrine cells were not organized but scattered, showing disturbing lobular architecture with dilated interlobular blood vessels surrounded by inflammatory cell infiltration. Moreover, a section of pancreatic cells of the group treated with GLB, showing ameliorated effects with restored islets of Langerhans and having moderately organized and intact glandular cells, is displayed in Figure 6C). Photomicrograph of pancreatic tissues from the group treated with 200 mg/kg of TME showing recovery of both endocrine (islets of Langerhans) and exocrine cells is exhibited in (Figure 6D). However, perilobular fibrosis is apparent. The group treated with 400 mg/kg of TME, which also has a better recovery with near-normal exocrine cells, restored islets of Langerhans compared to the diabetic control group (Figure 6E). Lastly, photomicrograph of the group treated with UFN showing recovered endocrine cells (islets of Langerhans) and exocrine cells is displayed in Figure 6F. The glandular cells appeared compact and intact with near-normal architecture.

### 2.6. Histopathological Analysis of Adipose Tissue of Diabetic Rats with TME (200 and 400 mg/kg) of B. eriophora and UFN Treatments

**Figure 7:**
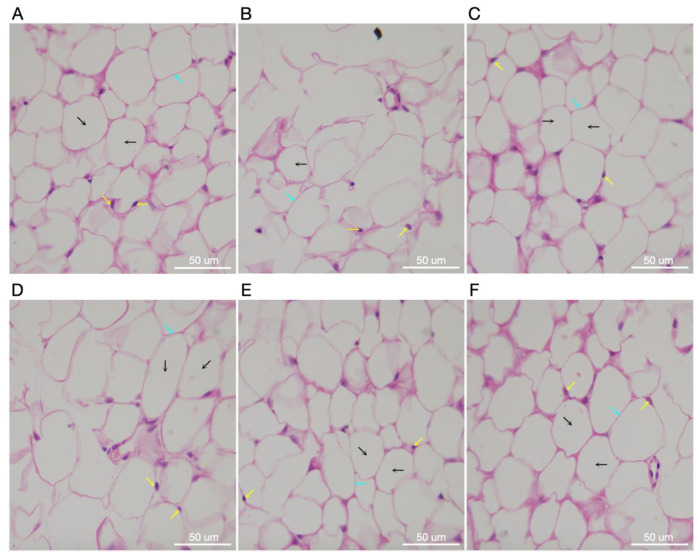
Photomicrographs of adipose tissue of controls and treated experimental animals. (**A**) The control group showed polygonal fat cells (black arrow) with a nucleus (yellow arrow) and distinct polygonal-shaped cells (light blue arrow). (**B**) The diabetic control group showed large-sized polygonal fat cells (black arrow) with scattered nuclei (yellow arrow) and distorted fat cells outlines (light blue arrow). (**C**) Showing recovered fat cells near to normal with GLB treatment. (**D**) Showing recovering adipose tissue fat cells with few nuclei and distorted polygonal fat cells after treatment with 200 mg/kg TME. (**E**) Shows apparent improved polygonal fat cells (black arrow) with well-shaped cellular outlines (light blue arrow). (**F**) Represents treatment with UFN also showing recovered adipose tissue fat cells (black arrow) with defined cellular outlines (blue arrow) but not as distinct as in the diabetic control group. H&E, ×1000. STZ; streptozotocin HFD; high-fat diet, STD GLB; standard glibenclamide, TME; total methanol extract, UFN; umbelliferone.

Figure 7 shows pathological changes in adipose tissue of STZ- and HFD-induced diabetic rats following treatments with TME and UFN. A microimage of the normal control’s adipose tissue shows polygonal fat cells with distinct parenchymal cells (Figure 7A). Conversely, the fat tissue of the diabetic control group displays distorted polygonal fat cells with no clear cell outlines of the adipocytes (Figure 7B). However, positive control, treated with standard GLB, represents adipose tissues with some degree of recovery from damage caused by STZ plus HFD and few lost cellular architectures, but appearing near normal (Figure 7C). Moreover, photomicrograph of adipose tissue in the group treated with 200 mg/kg of TME shows some degree of recovery of cells with fewer distorted polygonal adipose cells (Figure 7D). Conversely, the groups treated with the administration of 400 mg/kg of TME exhibits apparently normal fat cells within adipose tissue and has normally distributed nuclei (Figure 7E). Lastly, a micrograph of rats’ adipose tissues treated with UFN shows also an apparent normal cellular structure of adipose tissue, but not as distinct as in the group treated with 400 mg/kg of TME. From the foregoing, these results suggest that treatment with either TME or UFN appeared to restore some degree of normal adipose tissue architecture compared to the diabetic control group, but treatment with 400 mg/kg TME had a significant effect.

## 3. Discussion

Evidence from folk medicine has documented the use of traditional medicinal plants in the treatment of various diseases such as DM and obesity [36,37]. The use of these plants has gradually been introduced into modern medical practice because they are part of people’s cultural heritage. Hence medicinal plant sources are becoming alternatives in a sense of alleviating the oxidative damage caused by both disease and conventional drug treatments [38]. Studies have reported that *B. eriophora* extracts have antioxidant and anti-inflammatory potentials [25]. Reports have also documented the implication of oxidative stress in DM, therefore, plants such as *B. eriophora* with antioxidant activity can be another source of treatment approach [39]. It is always a task to design a method that will mimic human diseases to elucidate its potentials.

The present study was carried out to investigate the chemical composition of *B. eriophora,* evaluate its effects on blood glucose and lipid profile in STZ- and HFD- induced diabetic rats. The current study showed the isolation and identification of a coumarin compound from TME of *B. eriophora*. Based on, ^1^H, ^13^C, DEPT, HMQC and HMBC spectral data, the compound was identified as UFN (Supplementary data [S1–S6], Figure 1). This is the first report in the literature showing that the *B. eriophora* plant does contain this compound.

To evaluate the antidiabetic potential of *B. eriophora* TME and the identified compound, UFN, Type 2 DM model was developed by feeding rats with HFD for 8 weeks before the administration of STZ. The results revealed that HFD-fed rats gained weights that were significantly different from the control (Figure 2). The observation was consistent with other studies that used a similar method with regard to weight gain [40,41].

Therefore, the use of STZ + HFD has been reported to impair the secretions of insulin, which causes hyperglycemia and dyslipidemia, amongst other disorders [42,43,44]. Our study showed significant increases in blood glucose on the third day of STZ injection in rats fed with HFD. This finding is similar to that of many studies that used this model in investigating a disease condition that closely resembles human type 2 DM [42,45,46,47].

The increase in blood glucose level was significantly lowered upon treatment with TME of *B. eriophora* in a dose-dependent manner. Our observation could be related to the induction of oxidative stress by STZ + HFD [48], that was potentially abolished by treatment with TME of *B. eriophora* which was reported to possess an antioxidant property [28]. According to this report, TME of *B. eriophora* was found to alleviate oxidative stress in rats’ tissues. Our results showed that by the 3rd week of treatment with TME of *B. eriophora*, the blood glucose level in the treated diabetic group was found to be the same as the control group. Glycemic control exhibited by TME of *B. eriophora* in this study may also be due to the protection of beta cells in intact pancreatic islets of Langerhans and enhanced insulin release from the remnant of the pancreatic endocrine cells. The efficacy of 400 mg/kg of TME of *B. eriophora* in controlling the blood glucose level in STZ- and HFD-induced diabetic rats was better than GLB, an insulin secretagogue employed in type 2 diabetic patients. The mechanism of action of GLB is primarily by blocking the ATP-sensitive channels, hence causing the release of insulin from the pancreatic beta cells [49]. However, the effect of GLB was similar to that observed from treatment with 200 mg/kg of TME.

Lipid profile disruption in the form of hyperlipidemia is an established risk factor for type 2 DM [50]. It is characterized by elevated levels of TC, TRG and LDL [51]. In addition, documented evidence revealed the presence of an increased lipid profile in STZ- and HFD-induced diabetic rats [52]. In the present study, we observed a significant rise in the levels of serum TC, TRG, and LDL, coupled with a decrease in HDL level. These observations could be related to enhancing free fatty acids mobilization from peripheral fat depots to serum. A phenomenon that is seen in DM [51]. Furthermore, the absence or reduced activity of insulin means that there is an increase in lipolysis and hence more free fatty acids are transported into the blood circulation [51]. Treatment with TME of *B. eriophora* significantly decreased TC, TG, and LDL levels as well, in a dose-dependent fashion, in STZ- and HFD-induced diabetic rats. Moreover, HDL levels were significantly elevated, which has a reciprocal relationship with TC.

In type 2 diabetic conditions, there is a disturbed glucose uptake usually resulting in hyperglycemia and consequently increased insulin secretion, with time, that could result in insulin resistance [53]. Hence, in response to the increased blood glucose and insulin resistance, the beta cells of the pancreas increase the secretion of insulin as a result to maintain normal glucose hemostasis. This phenomenon creates a situation of hyperinsulinemia. In the present study, we observed that the level of serum insulin was increased significantly by STZ + HFD. This finding was in line with previously published report [54]. However, TME significantly reduced insulin levels in a dose-dependent fashion. In addition, HOMA-IR was also significantly decreased in a similar manner by TME treatment. This indicate that TME has the potential to reduce insulin resistance and improve glucose metabolism.

Additionally, histopathological examination of pancreatic tissues in STZ- and HFD-induced diabetic rats and their treatments were undertaken in the present study. Our results found damaged beta cells (islets of Langerhans) and diffused exocrine cells with the presence of perilobular fibrosis. The degradation and damage of pancreatic cells observed in this study were similar to a previously published study [23]. However, these pancreatic cellular abnormalities were restored upon the administration of TME of *B. eriophora* in a dose-related manner. The photomicrographs showed larger islets of Langerhans cells with improved cellular architecture. This, therefore, implied that TME of *B. eriophora* treatments attenuated the damage caused by STZ- and HFD-induced changes.

Furthermore, the histological examination of adipose tissues in STZ- and HFD-induced diabetes revealed distorted polygonal cells without parenchymal cell outlines. Once again, treatment with TME of *B. eriophora* markedly improved adipose tissue polygonal cells with apparent normal adipocytes. This corroborated the results seen with decreased lipid profile levels.

Several studies have indicated that UFN can improve both hyperglycemia and hypertriglyceridemia [55,56,57]. Moreover, UFN has been documented to possess antidiabetic activity by several researchers [58,59]. From the foregoing, we hypothesized that UFN described as an antioxidant could have the potential to ameliorate STZ- and HFD-induced damage of beta cells of Langerhans islets via alleviating oxidative stress [57]. Our results, therefore, are in total agreement with these aforementioned studies. Moreover, treatment with 50 mg/kg UFN significantly decreased the levels of TC, TG and LDL in STZ- and HFD-induced diabetic rats. These findings were also in line with previously published reports [60,61]. These studies revealed that the isolated UFN from banana flower and *Salvadora indica*, was able to reduce and improve lipid profiles in chemically induced diabetic animals. From the histopathological examination, oral treatment with UFN has restored both pancreatic and adipose tissue damage that was induced by STZ + HFD. Hence, a significant improvement in both glycemic and lipid profile control was observed with UFN treatment.

## 4. Materials and Methods

### 4.1. General Procedures and Chemicals

NMR spectra were obtained using Avance 400 NMR spectrometer (^1^H NMR: 400 MHz and ^13^C NMR: 100 MHz, Bruker, Switzerland). Silica gel column chromatography (SCC) was carried out on silica gel 60 (Sigma-Aldrich, Darmstadt, Germany). Diaion-HP-20 (Sigma-Aldrich, Darmstadt, Germany) and silica gel with F254 plates (Sigma-Aldrich, Darmstadt, Germany) were used in preparative thin layer chromatography (PTLC). Reversed-phase column chromatography (RPCC) was performed on C18-reversed-phase silica gel for column chromatography (Sigma-Aldrich, Darmstadt, Germany). Visualization was detected using 10% vanillin-sulfuric acid in ethanol with a hotplate (150 ^°^C). High-performance liquid chromatography (HPLC) apparatus (Agilent, 1200 series, Waldbronn, Germany) equipped with a degasser, autosampler, quaternary pump and PDA detector and Discovery® C18 (150 mm × 4.6 mm × 5 μm) column (Supelco, Bellefonte, Pennsylvania, USA) was used. An isocratic mode elution equipped with methanol:acetonitrile:water blend (*v/v/v*, 35:45:20) with a flow rate of 1.5 mL/min was used. The injection volume was 10 μL (70 mg/mL, sample in methanol). The chromatogram was monitored using Agilent ChemStation software ver.B.2.4.1. GLB (glibenclamide) and streptozotocin (STZ) were purchased from (Sigma-Aldrich, Taufkirchen, Germany). A glucose meter (Accu-Chek Guide) and glucose–oxidase–peroxidase reactive strips were purchased from (Accu-Chek, Roche Diagnostics GmbH, Mannheim, Germany) to estimate blood glucose levels. Kits for lipid profile analysis were purchased from (Labtest Diagnostica, Minas Gerais, Brazil). All other chemicals of analytical grade were purchased from standard commercial suppliers.

### 4.2. Plant Material

*B. eriophora* was collected from Al-Ahsa, Eastern Region, Saudi Arabia. The whole plant material was subjected to air-drying according to universal standard herbarium procedures. The plant was kindly identified by Eng. Mamdouh Shokry, director of El-Zohria botanical garden, Giza, Egypt. A voucher specimen (BE-April-2021) was kept in the Department of Pharmaceutical Sciences, College of Clinical Pharmacy, King Faisal University.

### 4.3. Extraction and Isolation

Air-dried powdered plant material (1 kg) was deeply extracted five times, with 10 L of methanol for 10 days at 25 °C with regular stirring. The resulting extracts were compiled and concentrated using a rotary evaporator to yield a dark green extract weighing 101 g. TME (50 g) was defatted using petroleum ether, where TME was suspended in 1 L of distilled water and partitioned with petroleum ether (5 times using 10 L) [62]. The resulting petroleum ether fractions were compiled and concentrated to dry to yield 14 g. The remaining mother liquor (36 g) was subjected to column chromatography using a Diaion HP-20 (1 kg) as a stationary phase, then eluted with pure water followed by 50% then 100% methanol to yield the fractions of water (named, BEI, 10 g), 50% methanol (named, BEII, 19 g) and 100% methanol (named, BEIII, 7 g). Based on TLC patterns, the BEII fraction (19 g) was subjected to SCC (500 g, using 5 L of chloroform:methanol: water with a composition ratio of (15:6:1) as mobile phase). The process has resulted in 5 main sub-fractions (BEII-1 to 5). Sub-fraction BEII-3 (2 g) was clear to contain a main spot with a large amount; hence it was selected for further purification. It was subjected to RPCC (250 g, applying gradient elution using methanol:water as mobile phase), to give the main spot as semi-pure compound which was further purified by PTLC and HPLC to yield pure umbelliferone (UFN) (200 mg).

### 4.4. Animals

Male Wistar rats weighing 195–210 g (8–10 weeks old) were used for this study. They were housed in line with the conventional protocol of natural photoperiod, consisting of 12 h light and 12 h darkness throughout the study. They were allowed constant access to food and water throughout the study. Animal care and experimental procedures were carried out in accordance with the approved guidelines of the Research Ethics Committee (with protocol ID: KFU-REC/2021-OCT-EA00012) at King Faisal University, Saudi Arabia.

### 4.5. Experimental Protocol

Initially, animals were divided randomly into two groups. The control group consisted of 6 rats, fed with commercial feed (normal rat chow), while the high-fat diet and STZ (HFD-STZ) group, which consisted of 30 rats, was fed with HFD for 8 weeks according to the referred methods with some modifications [63,64,65,66]. HFD was prepared in the lab with 50% normal rat chow, casein protein purchased as CaseinFX, 100% Casein Micellar Protein (from ALLMAX Nutrition), lard fat from Pure butter ghee 99.8%, butter fat from Almarai, Saudi Arabia, sucrose also purchased locally. The ingredients of commercial feed and HFD are shown in Table 1 and Table 2, respectively.

**Figure 8:**
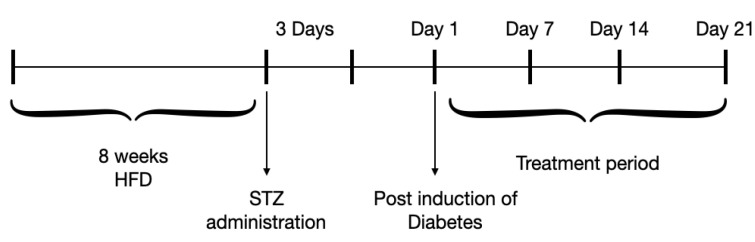
Schematic diagram for the induction of DM and treatment procedures in the experimental animals.

Following 8 weeks of HFD, the 30 animals were divided into five groups as shown in Table 3. Then, overnight-fasted animals were administered intraperitoneally (i.p.) with a single dose of 40 mg/kg STZ, prepared in a 0.1 M cold citrate buffer (pH 4.5), and monitored for 3 days as previously described [67,68,69]. A blood glucose level of > 250 mg/dL was confirmed as a state of hyperglycemia. The respective treatments of the different five diabetic groups were for 21 days as shown in Figure 8. Dosages were chosen according to previous experiments using TME of plants [23,70].

Blood samples were collected after an overnight fast via the tail vein for the measurement of blood glucose levels weekly, by using glucose–oxidase–peroxidase reactive strips (Accu-Chek Guide, Roche Diagnostics GmbH, Mannheim, Germany). An automated chemistry analyzer (Merck, Wiesbaden, Germany) and LABTEST kits including Cholesterol liquiform, Triglycerides liquiform, HDL liquiform, and LDL liquiform from (Labtest Diagnostica, Minas Gerais, Brazil) were used to measure lipid parameters such as serum total cholesterol, TRG, HDL cholesterol, and LDL cholesterol, respectively. Blood samples for insulin concentration determination were collected via cardiac puncture, and serum was collected and stored in a −85 °C freezer until analyzed. Insulin levels were measured in serum (μIU/mL) using a Cobas E 411 analyzer (Roche Diagnostics GmbH, D-68298 Mannheim, Germany), a fully automated analyzer that uses a patented ElectroChemiLuminescence (ECL) technology and analyzed as described by a published study [71]. HOMA-IR was calculated according to the formula: fasting insulin (µIU/mL) × fasting glucose (mg/dL)/405 [72]. The respective treatments of the different five diabetic groups were given orally and continued for 3 weeks, after which all rats were euthanized, and pancreatic and adipose tissues were harvested for a histopathology examination.

### 4.6. Histopathological Preparation and Examination

Pancreatic and adipose tissues of all the treated groups were harvested and fixed in a 10% buffered formaldehyde solution. After treatment for dehydration in alcohol, sections having 4μm thickness were cut and stained with hematoxylin and eosin (Merck, Darmstadt, Germany) and histopathological analysis of the resulting slides were carried out as previously described [73]. The slides were examined using a light microscope (Olympus, Tokyo, Japan). Photomicrographs were digitally captured using a high-resolution color digital camera (Olympus, Tokyo, Japan) adapted to the microscope and connected to a computer.

### 4.7. Statistical Analysis of Data

Data is hereby presented as mean ± standard deviation (SD) and statistical analysis was performed using GraphPad Prism software ver.8.2 (San Diego, CA, USA). Intergroup comparisons were performed by two-way analysis of variance (ANOVA) and differences between the groups were measured using Tukey’s multiple comparisons test. Statistically significant was taken as *p* < 0.01.

## 5. Conclusions

In summary, the present study indicated that TME of *B. eriophora* exhibited significant hypoglycemic and hypolipidemic effects in STZ- and HFD-induced diabetic rats. Insulin levels were reduced, with a consequent decrease in HOMA-IR with TME treatments. We also showed that pancreatic tissues responsible for insulin production were protected from damage, as was seen in STZ + HFD treated group. Furthermore, adipose tissue cellular structure damage, was ameliorated by *B. eriophora* TME administration. Additionally, UFN, the isolated compound, demonstrated similar characteristics, confirming that it has potential antidiabetic and antilipidemic properties. The current study hereby confirms the fact that UFN improves beta-cell functioning by attenuating pancreatic tissue oxidative damage. This subsequently enhances insulin secretion which is responsible for maintaining blood glucose and lipid profiles.

## Figures and Tables

**Table 1:** Ingredients (%/100 g) of commercial feed.

Ingredients of Commercial Feed	Per 100 g
Crude protein	14.5%
Fiber	4%
Fat	2.5%
Calcium	1%
Potassium	0.5%
Sodium	0.25%
Copper	6 ppm
Selenium	260 ppm
Vitamin A	8500 IU
Vitamin D	650 IU

**Table 2:** Ingredients (%/100 g) of high-fat diet.

Ingredients of High-Fat Diet	Per 100 g
Normal rat chow diet	50%
Lard and Fat oil	20%
Casein protein	10%
Sugar	20%

**Table 3:** Groups and their respective treatments.

Groups	Treatments
Negative control Diabetic control	No treatment (only distilled water)
Positive control STD GLB	6 mg/kg b.w. of standard GLB dissolved in carboxymethylcellulose (CMC) sodium orally once a day
TME 200	200 mg/kg b.w. of TME suspended in sterile water once a day
TME 400	400 mg/kg b.w. of TME suspended in sterile water once a day
UFN 50	50 mg/kg b.w. of UFN in distilled water once a day

## Data Availability

Not applicable.

## Supplementary Materials

The following supporting information can be downloaded at: https://www.mdpi.com/article/10.3390/molecules27206941/s1, Supplementary S1–S6: 1D- and 2D- NMR spectroscopic data of pure isolated compound umbelliferone.

## Sample Availability

Samples of the compounds are available from the authors.
