# Supplementary material for: Investigation of the Chemical Composition, Antihyperglycemic and Antilipidemic Effects of Bassia eriophora and Its Derived Constituent, Umbelliferone on High-Fat Diet and Streptozotocin-Induced Diabetic Rats
Source: Molecules. 2022 Oct 16;27(20):6941. doi: 10.3390/molecules27206941 (PMC9611308; doi:10.3390/molecules27206941)
Supplement: Supplementary file 1 [file molecules-27-06941-s001.zip › molecules-1945587-supplementary.pdf]

Manuscript ID
molecules-1945587

Title

Investigation of the chemical composition, antihyperglycemic and antilipidemic effects of Bassia eriophora and its derived constituent, Umbelliferone on high fat diet-streptozotocin induced diabetic rats

Supplementary-S1-S6: 1D- and 2D- NMR spectroscopic data of pure isolated compound umbelliferone

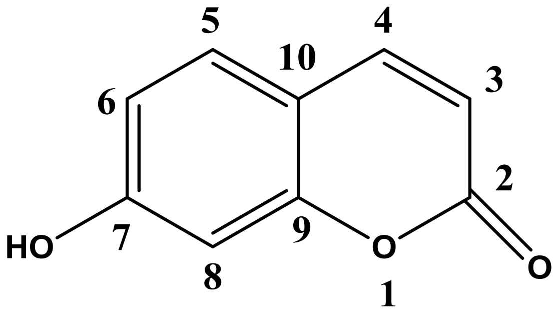

Structure of pure isolated umbelliferone .

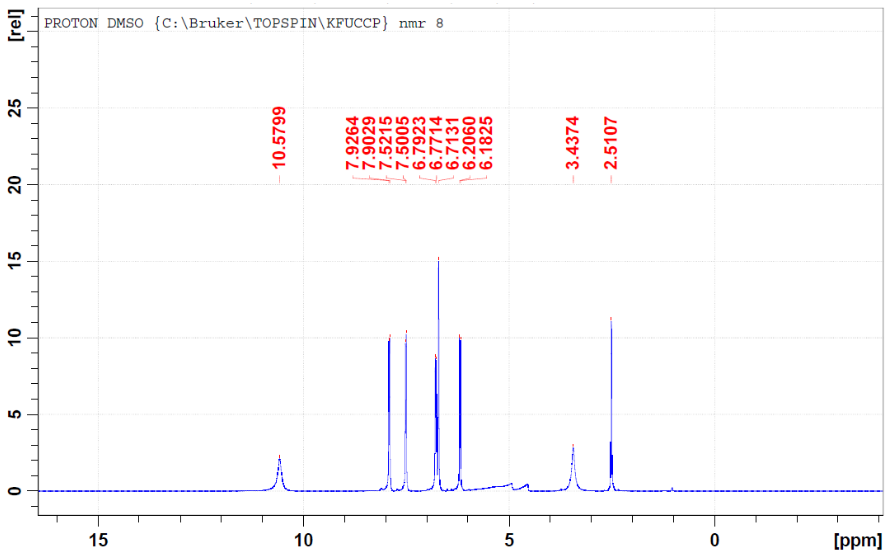

Figure S1 : 1H-NMR full spectrum of umbelliferone (400 MHz, DMSO-*d6*)

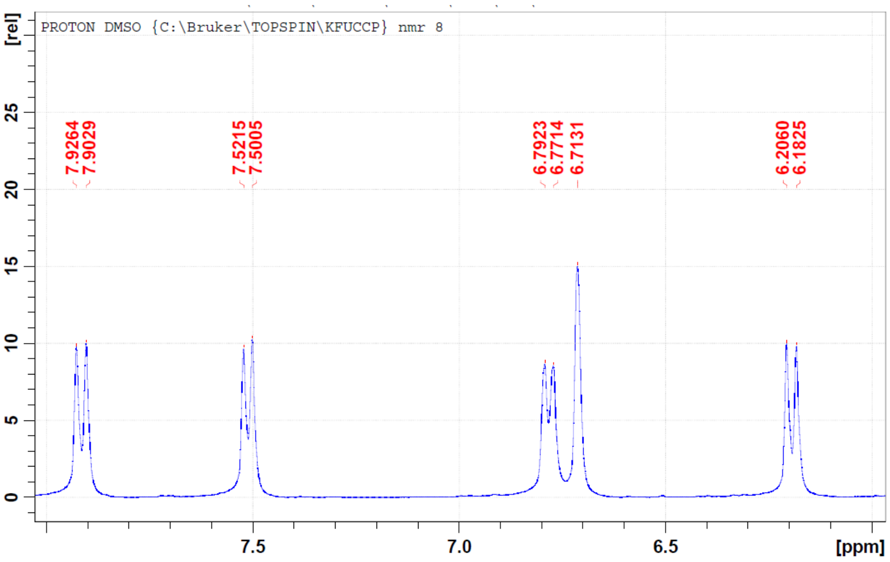

Figure S2: ^{1}H-NMR expanded spectrum of umbelliferone (400 MHz, DMSO-*d6*)

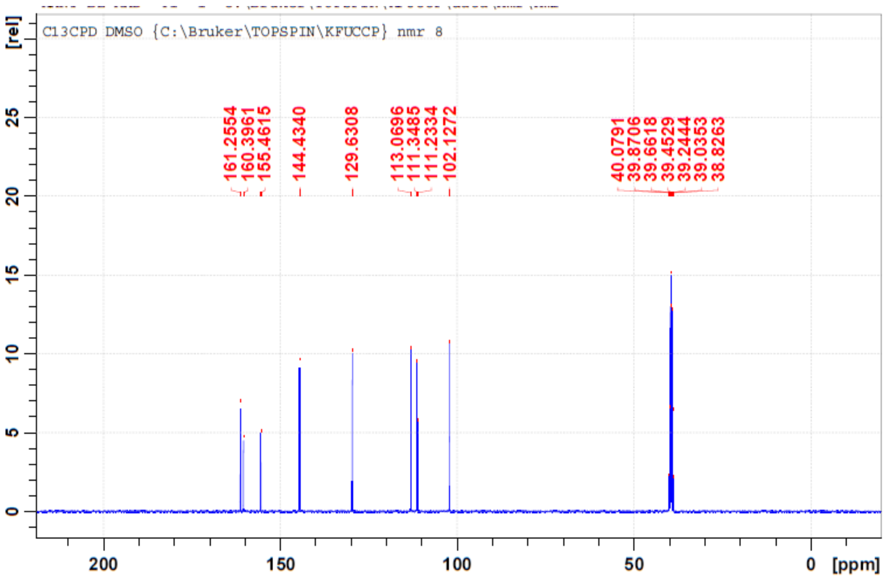

Figure S3: ^{13}C-NMR spectrum of umbelliferone (100 MHz, DMSO-*d6*)

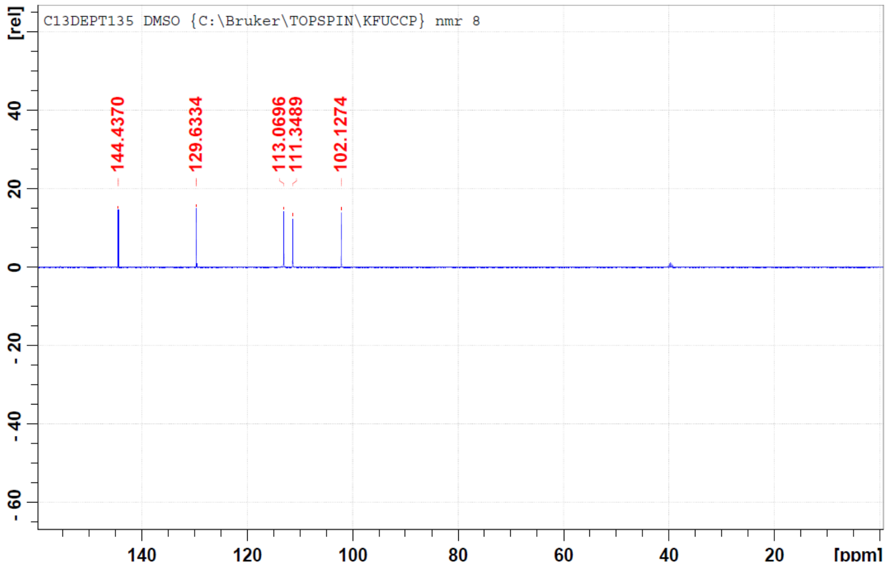

Figure S4 : DEPT spectrum of umbelliferone (100 MHz, DMSO-*d6*)

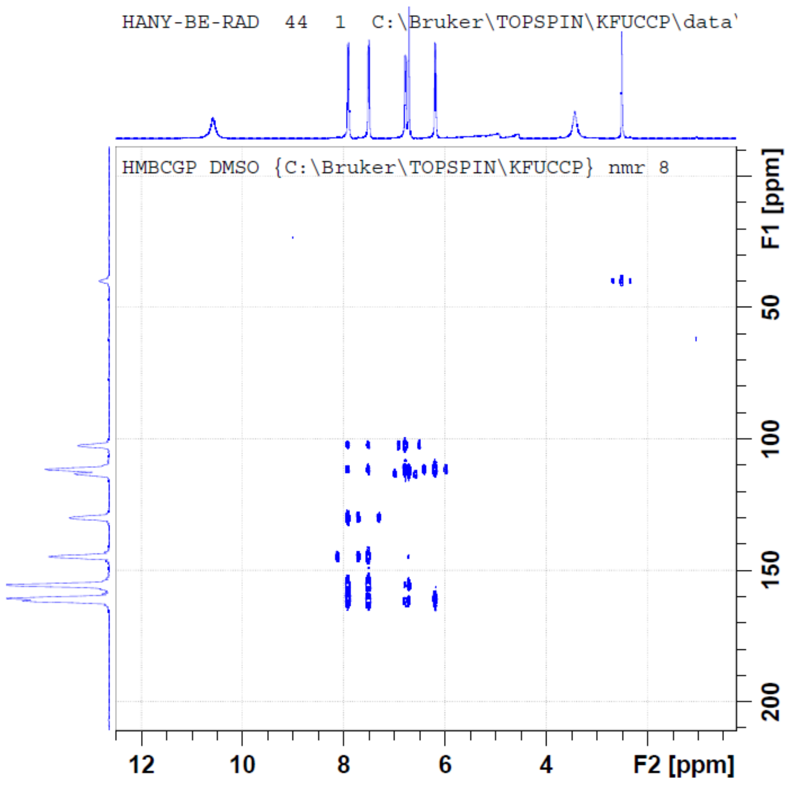

<u>**Figure S5 :HMBC spectrum of umbelliferone (400 MHz, DMSO-d6)**</u>

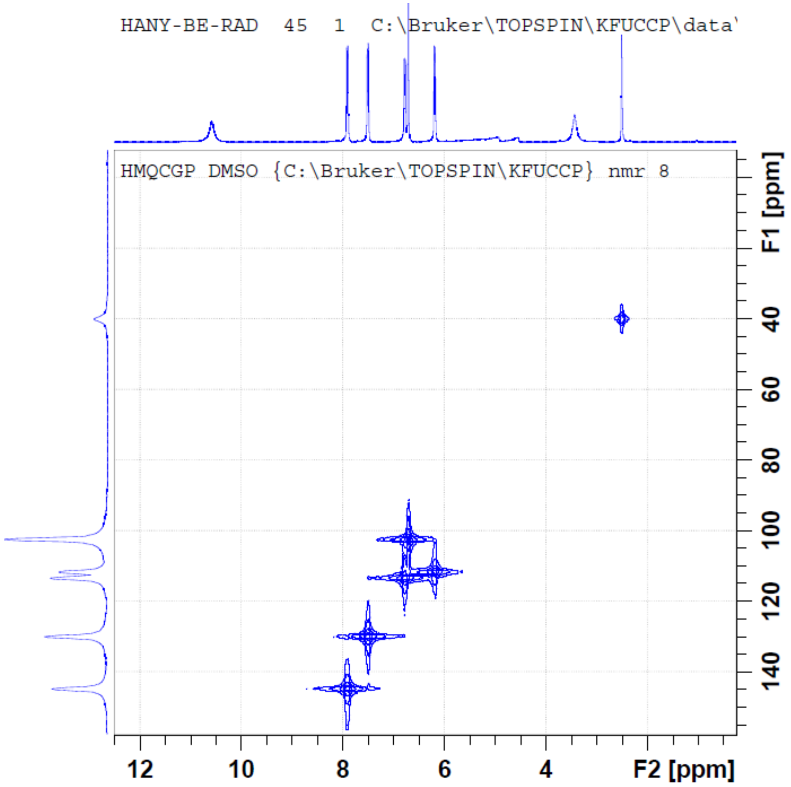

<u>**Figure S6 : HMQC spectrum of umbelliferone (400 MHz, DMSO-d6)**</u>
